# Protective Effects of Purple Rice Husk against Diabetic Nephropathy by Modulating PGC-1α/SIRT3/SOD2 Signaling and Maintaining Mitochondrial Redox Equilibrium in Rats

**DOI:** 10.3390/biom11081224

**Published:** 2021-08-17

**Authors:** Orawan Wongmekiat, Narissara Lailerd, Anongporn Kobroob, Wachirasek Peerapanyasut

**Affiliations:** 1 Renal Physiology Unit, Department of Physiology, Faculty of Medicine, Chiang Mai University, Chiang Mai 50200, Thailand; wachirasek.pee@mahidol.ac.th; 2 Nutrition and Exercise Unit, Department of Physiology, Faculty of Medicine, Chiang Mai University, Chiang Mai 50200, Thailand; narissara.lailerd@cmu.ac.th; 3 Division of Physiology, School of Medical Science, University of Phayao, Phayao 56000, Thailand; anongporn.ko@up.ac.th

**Keywords:** diabetes, kidney, mitochondria, *Oryza sativa*, oxidative stress, rice husk

## Abstract

Diabetic nephropathy (DN) is the primary cause of end-stage renal disease worldwide. Oxidative stress and mitochondrial dysfunction are central to its pathogenesis. Rice husk, the leftover from the milling process, is a good source of phytochemicals with antioxidant activity. This study evaluated the possible protection of purple rice husk extract (PRHE) against diabetic kidney injury. Type 2 diabetic rats were given vehicle, PRHE, metformin, and PRHE+metformin, respectively, while nondiabetic rats received vehicle. After 12 weeks, diabetic rats developed nephropathy as proven by metabolic alterations (increased blood glucose, insulin, HOMA-IR, triglycerides, cholesterol) and renal abnormalities (podocyte injury, microalbuminuria, increased serum creatinine, decreased creatinine clearance). Treatment with PRHE, metformin, or combination diminished these changes, improved mitochondrial function (decreased mitochondrial swelling, reactive oxygen species production, membrane potential changes), and reduced renal oxidative damage (decreased lipid peroxidation and increased antioxidants). Increased expression of PGC-1α, SIRT3, and SOD2 and decreased expression of Ac-SOD2 correlated with the beneficial outcomes. HPLC revealed protocatechuic acid and cyanidin-3-glucoside as the key components of PRHE. The findings indicate that PRHE effectively protects against the development of DN by retaining mitochondrial redox equilibrium via the regulation of PGC-1α-SIRT3-SOD2 signaling. This study creates an opportunity to develop this agricultural waste into a useful health product for diabetes.

## 1. Introduction

Diabetes mellitus is currently a serious public health problem that affects millions of individuals worldwide. Diabetic nephropathy (DN), also called diabetic kidney disease, is one of the most important long-term complications in terms of morbidity and mortality for individual patients with diabetes. It is also accepted as the leading cause of end-stage renal disease in many countries around the world [1]. The latest report by the International Diabetes Federation (IDF) shows that half a billion people worldwide are diabetic, and more than 80% of cases of end-stage renal disease are caused by diabetes [2]. Thailand is among the countries in Asia with a high prevalence of diabetes. Currently, diabetes in the Thai population is estimated at 4.2 million cases, or 7–8% of the population, of which about 44% are experiencing diabetic kidney disease [2,3]. Global records, including Thailand, indicate that the number of diabetic patients who require renal replacement therapy has progressively increased over the last two decades [4]. The issue not only produced a significant burden on economics but also caused psychosocial problems in terms of reduced quality of life and suffering of the patients. Importantly, data from the Thai National Health Examination Survey and the IDF atlas reveal that diabetes-related deaths are approximately 12–15% of total deaths in Thailand [2,5,6]. Finding strategies to prevent or slow down the onset and progression of kidney disease in people with diabetes remains an important and necessary task.

Rice is the seed of the grass species *Oryza sativa*. It is the agricultural commodity with the third highest worldwide production. Rice is the most widely consumed staple food in over half of the world’s population, particularly in Asia and Africa [7]. Though rice can be cultivated worldwide, more than 90% is grown in Asia. Nowadays, the trend of health promotion with natural supplements is becoming increasingly popular. Rice, especially colored rice, is one of the popular choices, as it contains nutrients, vitamins, and phytochemicals. There is a variety of colored rice, including brown, red, and purple (or black) rice. Among these colored rice, purple rice has received the most considerable interest due to its anthocyanin content, which is higher by weight than that of other colored grains [8]. Studies have shown several health benefits of extracts from diverse parts of purple rice, i.e., rice grain and rice bran [9,10], and thus greatly increased the demand for this colored rice. Rice husks, a major organic source of waste of the rice milling process, are also accumulated. These wastes are often removed by burning, causing air pollution that directly affects climate change, quality of life, and human health. Therefore, turning these agricultural wastes into valuable stuffs is of interest and becomes challenging research. A study regarding rice husk, particularly from pigmented rice, indicated that rice husk is a good source of phytochemicals with antioxidant activity and suggested that it may have a great potential to turn into functional food or nutraceuticals to prevent diseases related to oxidative stress [11]. Recently, extract from purple rice husk has been shown to be a potent anticancer agent without any toxicity [12].

Mitochondria is well recognized as a major source of reactive oxygen species (ROS) production in the body, and thus, the maintenance of mitochondrial oxidative balance is essential. The peroxisome proliferator-activated receptor gamma coactivator 1-alpha-sirtuin 3-superoxide dismutase 2 (PGC-1α-SIRT3-SOD2) axis plays a significant role in the regulation of mitochondrial redox homeostasis [13,14]. PGC-1α is a nuclear-encoded transcriptional coactivator that regulates the expression of several nuclear-encoded mitochondrial proteins, including mitochondrial antioxidant and biogenesis genes [14]. SIRT3, a downstream target of PGC-1α, is a major mitochondrial deacetylase that directly deacetylates and activates numerous mitochondrial proteins, including the major mitochondrial antioxidant enzyme SOD2 [13,14,15]. SIRT3 is also being accepted as a global regulator playing a multifaceted role in the mitochondrial adaptive response to stress and is currently indicated as a new target for therapy aimed at improving end-organ damage and survival [14,15].

As reactive oxygen species (ROS) production, oxidative stress generation, and, particularly, mitochondrial dysfunction are central to the pathogenesis of diabetic nephropathy [1,16], it is assumed that purple rice husk extract (PRHE) may be able to prevent or reduce diabetes-induced renal deterioration. Herein, we examined the renoprotective potential of PRHE in a rat model of high-fat diet/streptozotocin-induced type 2 diabetes (T2DM) and explored whether the modulation of the PGC-1α-SIRT3-SOD2 axis contributes to protection by PRHE.

## 2. Materials and Methods

### 2.1. Plant Materials and Preparation of PRHE

Thai purple rice (*Oryza sativa* L. var. Indica) cv. Kum Doisaket was first identified by Associate Professor Dr. Dumnern Karladee, Faculty of Agriculture, Chiang Mai University, Thailand (Voucher Specimen No.: Rawiwan_001) and planted at Mae Hia Agricultural Research, Faculty of Agriculture, Chiang Mai University. After harvested, the purple rice was confirmed again by comparing it to the known specimen identity deposited at the Faculty of Pharmacy, Chiang Mai University (Herbarium No.: 023252). Then, the rice husk was separated from the rice paddy and soaked in 0.1% hydrochloric in absolute methanol for 48 h at room temperature. The procedure was repeated twice, and the entire solution was filtered through Whatman No. 1 filter paper. The supernatant was evaporated under reduced pressure and lyophilized to obtain PRHE, which was kept in tight-sealed dark containers and stored at −20 °C for further studies.

### 2.2. Phytochemical Analysis, Quantification of Bioactive Compounds, and Evaluation of Antioxidant Capacity of PRHE

PRHE was initially examined for its major phytochemical constituents. The total amounts of phenolic, flavonoid, and anthocyanin were measured by Folin–Ciocalteu, aluminum trichloride, and pH differential methods, respectively, according to procedures described previously [17,18]. The phenolic acids and anthocyanins in PRHE were further identified and quantified by high-performance liquid chromatography (HPLC) using conditions and external standards as previously published [6]. 2,2′-Azino-bis(3-ethylbenzothiazoline-6-sulphonic acid) (ABTS) radical cation decolorization and 2,2-diphenyl-1-picryl-hydrazyl (DPPH) radical scavenging capacity assays were performed to evaluate the antioxidant capacity of PRHE according to the standard method previously described [19].

### 2.3. Animals and Experimental Protocols

Male Wistar rats weighing 150–200 g (National Laboratory Animal Center, Mahidol University, Salaya, Thailand) were housed in a controlled environment (24 ± 1 °C, 55 ± 5% humidity, 12 h light/dark cycle) with free access to food and water. All procedures complied with the guidelines established by the National Research Council of Thailand and were approved by the Animal Care and Use Committee of the Faculty of Medicine, Chiang Mai University (Project Number: 41/2559).

After acclimatization, rats were assigned to receive a normal diet (ND, *n* = 6) or a high-fat diet (HFD, *n* = 24). The diet composition is shown in the Appendix A (Table A1). Diabetes was induced in the HFD-fed rats two weeks later by a single intraperitoneal injection of streptozotocin (35 mg/kg in 0.1 M sodium citrate buffer, pH 4.5) [20], while the ND-fed rats received an equal amount of sodium citrate buffer. Two weeks after injection, rats with fasting blood glucose ≥250 mg/dL without hypoinsulinemia were considered diabetes and were included in the study.

Diabetic rats were randomly divided into 4 groups (*n* = 6 each): diabetic control group received vehicle (DMV), diabetic positive control group treated orally with metformin 50 mg/kg/day (DMM), diabetic group treated orally with PRHE 300 mg/kg/day (DME), and diabetic group treated with a combination of metformin and PRHE (DMME). The dose of PRHE was based on our preliminary results showing its efficacy in improving renal function together with lowering blood glucose and insulin resistance appropriately (Appendix A, Table A2). All diabetic rats were maintained on HFD for a further 12 weeks, whereas those in the nondiabetic group received a normal diet and vehicle (NDV). The 24 h urine samples were collected at the end of the study using metabolic cages, and blood and kidney tissues were collected thereafter under thiopental anesthesia (60 mg/kg, i.p.). Parts of the kidneys were rapidly taken for mitochondrial and histopathological studies. The remainders were snap-frozen in liquid nitrogen and stored at −80 °C for further analysis.

### 2.4. Biochemical Assays

#### 2.4.1. Determinations of Metabolic Indexes

Fasting blood glucose, cholesterol, and triglycerides were measured by enzymatic assay kits (ERBA Diagnostics Inc., Miami, FL, USA). Plasma insulin was quantified using ELISA kit (Millipore, Burlington, MA, USA). The Homeostasis Model Assessment for Insulin Resistance was calculated (HOMA-IR = fasting insulin level (ng/mL) × fasting glucose (mg/dL)/405.1) as described previously [21].

#### 2.4.2. Determinations of Renal Functions

Serum creatinine, urine creatinine, and urine microalbumin were analyzed using the AU480 Chemistry Analyzer (Beckman Coulter, Inc., Brea, CA, USA). Glomerular filtration rate (GFR) was estimated by calculation of the creatinine clearance using a standard clearance formula (the ratio of creatinine in urine/serum and the volume of urine produced).

#### 2.4.3. Determinations of Renal Oxidative Stress

The supernatant from kidney homogenate was used for oxidative stress assays. Malondialdehyde (MDA) was estimated using the TBARS assay kit (Cayman Chemical, Ann Arbor, MI, USA), glutathione (GSH) was assayed using the QuantiChrom™ Glutathione Assay Kit (Bioassay Systems, Hayward, CA, USA), and superoxide dismutase (SOD) and glutathione peroxidase (GPx) were determined using Calbiochem^®^ Assay Kits (Merck Millipore, Darmstadt, Germany). All analyses were performed according to the manufacturer’s instructions.

#### 2.4.4. Determinations of Mitochondrial Functions

Kidney mitochondria were isolated by differential centrifugation, and the mitochondrial proteins obtained were used to study mitochondrial function according to the protocols previously published [22]. Mitochondrial ROS production was determined using a fluorogenic dye dichlorofluorescin diacetate (DCFDA). ROS level was assessed from the fluorescent product and expressed in arbitrary units of fluorescent intensity. An increasing intensity indicates an increase in mitochondrial ROS production. Mitochondrial membrane potential change was assessed by staining mitochondria with a lipophilic cationic fluorescence JC-1 dye. The fluorescence intensity of JC-1 aggregate (red fluorescence) and monomer (green fluorescence) was measured, and a decrease in the red/green fluorescence intensity ratio implies the loss of membrane potential. Mitochondrial swelling was determined through changes in the absorbance of the mitochondrial suspension at 540 nm, where a decrease in absorbance intensity indicates the swelling of mitochondria.

### 2.5. Histopathological Examinations

Kidney tissues were routinely processed for light and electron microscopic examinations using the protocols previously described [13]. For the light microscopic study, a paraffin section of 4 μm was stained with hematoxylin and eosin (H&E) and examined by an unbiased pathologist using a Leica DM750 photomicroscope (Leica Microsystems, Heerbrugg, Switzerland). For electron microscopy, renal cortical sections embedded in Epon resin were stained with uranyl acetate and lead citrate, then examined using a JEM-2200 FS transmission electron microscope (JEOL, Tokyo, Japan).

The formalin-fixed paraffin-embedded kidney tissues were also used for CD34 immunofluorescence staining. Kidney sections (4 μm) were deparaffinized in xylene then rehydrated through an ethanol series. Antigen retrieval with 10 mM sodium citrate buffer (pH 6.0) was performed using a hot water bath for 10 min. The sections were permeabilized in PBST containing 1% BSA and 0.4% Triton X-100 for 10 min, followed by blocking with 5% BSA for 30 min at room temperature. Next, the slides were incubated with anti-CD34 antibodies (US Biological Life Sciences, Salem, MA, USA) for 2 h at room temperature then overnight at 4 °C. Fluorescence was observed under an inverted fluorescence microscope (Nikon, Eclipse Ts2, Nikon Instruments Inc., Melville, NY, USA).

### 2.6. Western Blot Analysis

Renal cortical tissues were homogenized in lysis buffer containing a protease inhibitor cocktail (Sigma-Aldrich, St. Louis, MO, USA), and protein concentrations were measured using the Bio-Rad protein assay kit (Bio-Rad Laboratories, Hercules, CA, USA). The protein sample was subjected to 10% sodium dodecyl sulfate–polyacrylamide gel electrophoresis (SDS-PAGE) and transferred to a polyvinylidene fluoride (PVDF) membrane, where it was blocked and probed with primary antibodies against peroxisome proliferator-activated receptor gamma coactivator 1-alpha (PGC-1α), sirtuin 3 (SIRT3), acetylated superoxide dismutase 2 (Ac-SOD2), superoxide dismutase 2 (SOD2), and β-actin (Cell Signaling Technology, Danvers, MA, USA), followed by corresponding horseradish peroxidase-conjugated secondary antibodies (Millipore Sigma, Darmstadt, Germany). Protein band densities were detected by the ChemiDoc^TM^ Touch Imaging System (Bio-Rad Laboratories, Inc., Philadelphia, PA, USA) and quantified using ImageJ software (National Institute of Health, Bethesda, MD, USA).

### 2.7. Statistical Analyses

Results are presented as the mean ± SD. Values related to phytochemical analysis and the antioxidant capacity of PRHE were obtained from three independent experiments, where each experiment was repeated three times. Comparisons between groups were assessed using One-way ANOVA followed by Fisher’s least-significant difference (SPSS 20.0, IBM Corporation, Armonk, NY, USA), and the significance level was set at *p* < 0.05.

## 3. Results

### 3.1. PRHE Possesses Antioxidant Capacity and Contains Protocatechuic Acid and Cyanidin-3-Glucoside as Major Phenolic-Based Compounds

**Figure 1:**
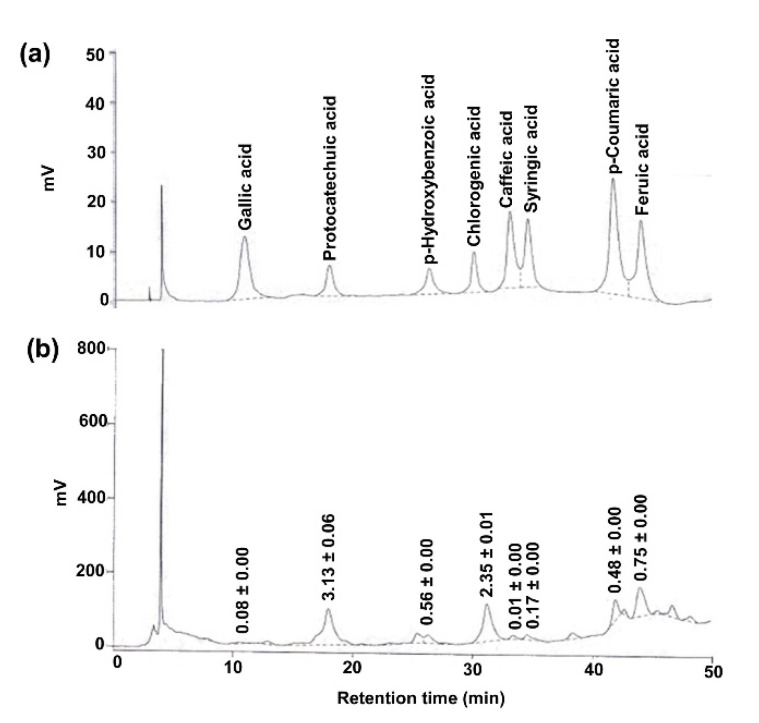
HPLC chromatogram at 280 nm showing major phenolic acids of purple rice husk extract. (**a**) Reference standards; (**b**) purple rice husk extract. The chromatographic peaks of the extract were confirmed by comparing their retention time and UV spectra with those of the reference standards. Numerical values denote the amount of individual compounds detected. Data (mean ± SD, *n* = 3) are expressed in mg/g of the extract.

**Figure 2:**
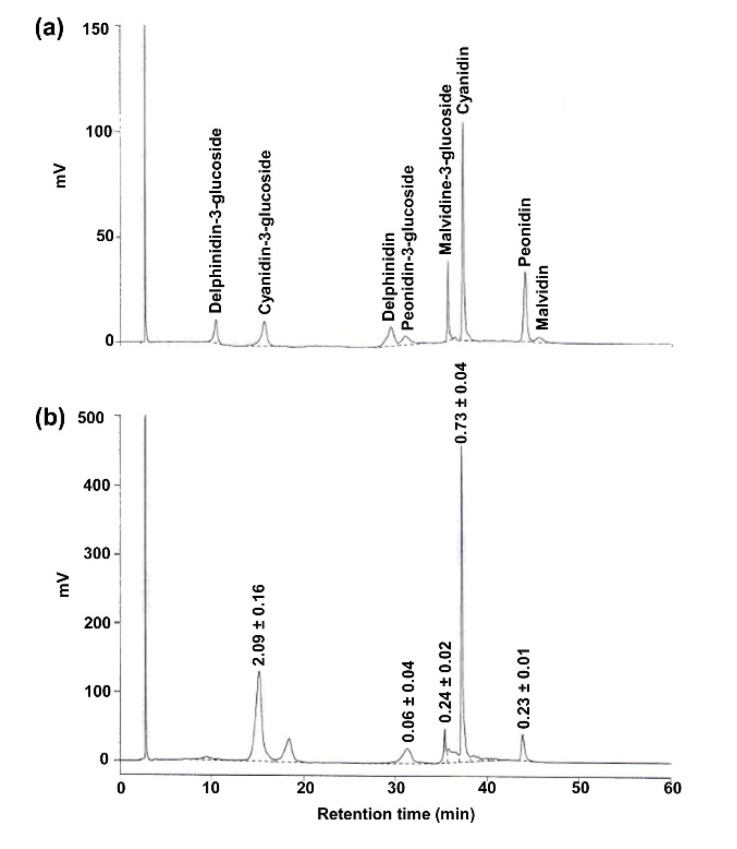
HPLC chromatogram at 520 nm showing major anthocyanins of purple rice husk extract. (**a**) Reference standards; (**b**) purple rice husk extract. The chromatographic peaks of the extract were confirmed by comparing their retention time and UV spectra with those of the reference standards. Numerical values denote the amount of individual compounds detected. Data (mean ± SD, *n* = 3) are expressed in mg/g of the extract.

Initial screening of phytochemical elements in PRHE revealed the presence of phenolics (98.66 ± 0.003 mg gallic acid equivalents/g extract), flavonoids (64.26 ± 0.002 mg quercetin equivalents/g extract), and anthocyanins (7.90 ± 0.030 mg cyanidin-3-glucoside equivalents/g extract). DPPH and ABTS radical scavenging assay demonstrated the antioxidant power of PRHE, being 12.42 ± 0.16 mg gallic acid equivalents/g extract and 152.96 ± 0.77 mM Trolox equivalents/g extract, respectively. HPLC further revealed a variety of phenolic acids (Figure 1) and anthocyanins (Figure 2), where protocatechuic acid (PCA) and cyanidin-3-glucoside (C3G) appeared to be the major phenolic acid and anthocyanin found in PRHE, respectively.

### 3.2. PRHE Improves Diabetes-Induced Metabolic Alterations

The diabetic control group (DMV) showed significant body weight gain along with increased visceral fat, serum cholesterol, and triglycerides compared to the nondiabetic group (NDV), though the energy intake was comparable (Table 1). A significant increase in fasting blood glucose and insulin, including HOMA-IR, was also observed in the DMV group compared to the NDV group. Diabetes-induced metabolic alterations were significantly diminished in the PRHE-treated group (DME). Interestingly, treatment with PRHE was as effective as metformin alone (DMM) or metformin plus PRHE (DMME).

### 3.3. PRHE Ameliorates Diabetes-Induced Renal Functional and Structural Impairments

**Figure 3:**
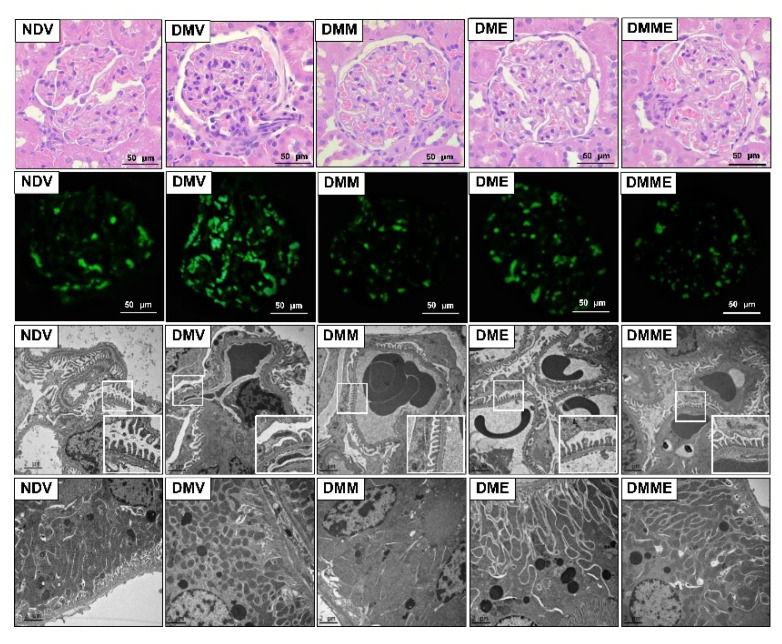
Photomicrographs of the kidney tissues following purple rice husk extract (PRHE) and metformin treatment. First panel shows kidney sections stained with hematoxylin and eosin (H&E, 40×). The second panel shows immunofluorescence staining of CD34 (magnification, 40×). The third and last panels show transmission electron micrographs of glomerulus and renal tubules, respectively (original magnification: 3000×). The boxed areas are magnified in the right lower panel. NDV: vehicle-treated normal group; DMV: vehicle-treated diabetic group; DMM: metformin-treated diabetic group; DME: PRHE-treated diabetic group; DMME: metformin plus PRHE-treated diabetic group.

Diabetic rats exhibited a significant increase in kidney weight/body weight ratio, serum creatinine, and microalbuminuria and a reduction in creatinine clearance (Table 2). These changes were significantly reduced, to a similar extent, when treated with PRHE, metformin, or both. In agreement with renal functions, kidney sections obtained from diabetic rats displayed perihilar focal segmental mesangial cell proliferation with mesangial area expansion and collagen deposits, together with a larger glomerular volume than normal rats (Figure 3, first panel). Immunofluorescence staining of CD34, a mesangial cell marker, also verified the presence of mesangial proliferation (Figure 3, second panel). Electron micrograph clearly showed glomerular basement membrane thickening with diffuse and extensive podocyte effacement (Figure 3, third panel), including the smaller size, abnormal shape, and decreased number of mitochondria within the proximal tubules (Figure 3, last panel). All these changes were significantly attenuated upon treatment with PRHE, metformin, or both.

### 3.4. PRHE Attenuates Diabetes-Induced Renal Oxidative Stress and Mitochondrial Dysfunction

**Figure 4:**
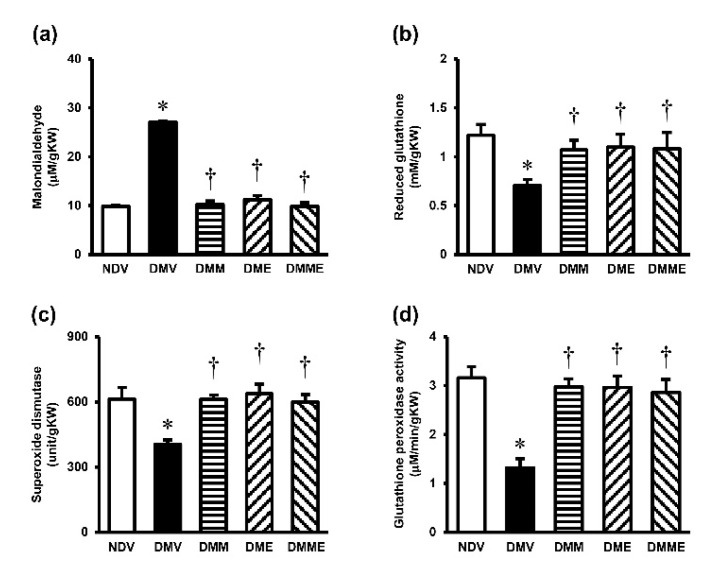
Effects of purple rice husk extract (PRHE) and metformin on renal oxidative stress indexes. (**a**) malondialdehyde; (**b**) reduced glutathione; (**c**) superoxide dismutase; (**d**) glutathione peroxidase. Values are the mean ± SD (*n* = 6). NDV: vehicle-treated normal group; DMV: vehicle-treated diabetic group; DMM: metformin-treated diabetic group; DME: PRHE-treated diabetic group; DMME: metformin plus PRHE-treated diabetic group; KW: kidney weight. * *p* < 0.05 vs. NDV, ^†^ *p* < 0.05 vs. DMV.

**Figure 5:**
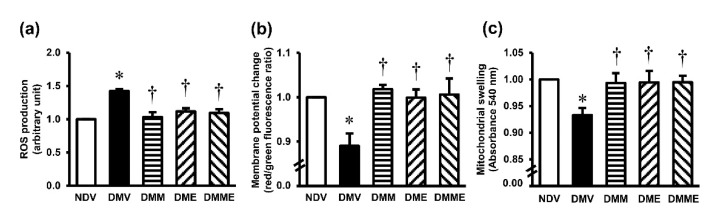
Effects of purple rice husk extract (PRHE) and metformin on kidney mitochondrial function. (**a**) ROS production; (**b**) membrane potential change; (**c**) mitochondrial swelling. Values are the mean ± SD (*n* = 6). NDV: vehicle-treated normal group; DMV: vehicle-treated diabetic group; DMM: metformin-treated diabetic group; DME: PRHE-treated diabetic group; DMME: metformin plus PRHE-treated diabetic group. * *p* < 0.05 vs. NDV, ^†^ *p* < 0.05 vs. DMV.

A significant increase in MDA but a decrease in GSH, SOD, and GPx were detected in the kidney tissues of diabetic rats (Figure 4). Treatment with PRHE and metformin and the combination regimen showed comparative efficacy to significantly attenuate these alterations. Diabetic rats also demonstrated a significant increase in mitochondrial ROS production, a decrease in mitochondrial membrane potential, and a swelling of the mitochondria (Figure 5). Supplementation of PRHE to the diabetic rats significantly restored all the changes. Metformin alone or in combination with PRHE showed similar results to PRHE treatment alone.

### 3.5. PRHE Modifies PGC-1α-SIRT3-Ac-SOD2-SOD2 Signaling Transduction

**Figure 6:**
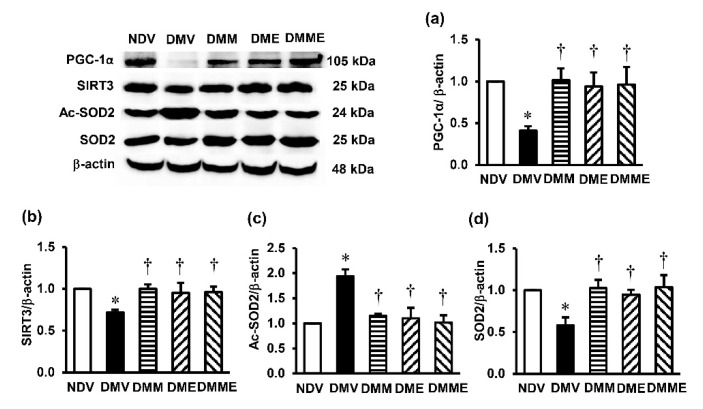
Effects of purple rice husk extract (PRHE) and metformin on renal cortical expression of (**a**) Peroxisome proliferator-activated receptor gamma coactivator 1-alpha (PGC-1α); (**b**) sirtuin 3 (SIRT3); (**c**) acetylated superoxide dismutase 2 (Ac-SOD2); (**d**) superoxide dismutase 2 (SOD2) and β-actin as a reference control. Values are the mean ± SD (*n* = 3). NDV: vehicle-treated normal group; DMV: vehicle-treated diabetic group; DMM: metformin-treated diabetic group; DME: PRHE-treated diabetic group; DMME: metformin plus PRHE-treated diabetic group. * *p* < 0.05 vs. NDV, ^†^ *p* < 0.05 vs. DMV.

To determine the signaling pathway involved in the benefits of PRHE on diabetic kidney injury, the protein expression of PGC-1α, SIRT3, Ac-SOD2, and SOD2 was determined. In the DMV group, the expression levels of PGC-1α, SIRT3, and SOD2 were significantly decreased compared to the NDV group, while the expression of Ac-SOD2 was significantly enhanced (Figure 6). These alterations were significantly reversed upon treated diabetic rats with PRHE. Similar results were observed in metformin monotherapy and in combination with PRHE.

## 4. Discussion

This study reveals the protection against nephropathy in T2DM by PRHE through antioxidant abilities and mitochondrial protection, possibly via its major phytochemicals, particularly PCA and C3G. Results suggest that modifications of PGC-1α/SIRT3/SOD2 signaling are linked to this protection.

A combination of a high-fat diet and streptozotocin injection was used to develop a rat model of T2DM in the present study. Feeding rats with a high-fat diet promotes the development of obesity and insulin resistance, while injection of streptozotocin selectively destroys pancreatic β-cells and, thus, impairs insulin secretion [20]. This model has been shown to replicate the natural history and metabolic characteristics of humans and is also suitable for pharmacological screening [20]. However, the reliability and appropriateness of the model depend on the number of pancreatic β-cells remaining, which is largely regulated by the dose of streptozotocin injection. A high dose of streptozotocin has been shown to critically damage pancreatic β-cell function, leading to insulin deficiency, which is considered to resemble T1DM rather than T2DM [23]. Regarding our model, hyperglycemia, hyperinsulinemia, insulin resistance (presented by increased HOMA-IR), and lipid abnormalities were all detected in the DMV group. These metabolic alterations are compatible with the well-recognized characteristics of T2DM, thus confirming the successful T2DM induction in the current study. We did not quantify the number of functioning pancreatic β-cells. However, the observations of hyperglycemia together with hyperinsulinemia in our diabetic rats implied that the number of intact pancreatic β-cells was sufficient to increase insulin secretion in response to high glucose, even though it could not overcome a progressive decline in insulin action caused by diabetes. Additionally, the degree of hyperglycemia and the dose of streptozotocin (35 mg/kg) used in our study corresponded to a previous report showing that streptozotocin at the dose of 30–40 mg/kg has a moderate effect on plasma glucose and on the islets of Langerhans at a cellular level [24]. Taken together, all evidence points towards the development of T2DM in the present study. Most importantly, our diabetic model also developed diabetic kidney injury, as indicated by accumulation of blood urea nitrogen and creatinine, reduced creatinine clearance, microalbuminuria, and histopathology that characterize diabetic nephropathy.

We found that diabetes-induced metabolic alterations were significantly diminished in the diabetic group given PRHE, suggesting the glucose-lowering ability of PRHE. To our knowledge, this is the first evidence showing antihyperglycemic potential in T2DM from the husk of purple rice, as a report is available only on its bran [25]. PCA and C3G, the main phytochemicals, may play a role in this action of PRHE. Previous publications showed the potential of PCA and C3G in reducing blood glucose, enhancing insulin sensitivity, and increasing glucose uptake, thereby improving glucose homeostasis in diabetes [26,27,28]. Studies also indicated that increased expression and translocation to the cell membrane of glucose transporters 1 (GLUT1) and 4 (GLUT4) contributed to the improvement of glucose tolerance by PCA and C3G [16,17,18]. The effectiveness on glycemic control by PCA and C3G was found to be comparable to metformin, a gold-standard antidiabetic drug, and was associated with the activation of AMPK [16]. Interestingly, our results also demonstrated a similar efficacy on metabolic improvement between PRHE, metformin, and PRHE+metformin. Metformin is an AMPK activator, and AMPK activation is recognized as the initial process for metabolic control by metformin [26]. It may be that PRHE (via PCA and C3G) stimulated AMPK and exerted its metabolic control through the same downstream signaling pathways as metformin; thus, no additional benefits were found in the combination therapy. Further study using cell culture experiments or the knockout mice model with an AMPK inhibitor or activator will be helpful to validate this possibility.

Our results showed that PRHE effectively protected against diabetic kidney injury. Because metabolic impairment was improved after PRHE treatment, it is likely that renal protection may result from glycemic control by PRHE. However, PRHE (particularly through PCA) may exert its benefits independent of glycemic control. This suggestion was based on a study using human mesangial cells exposed to high glucose in the presence or absence of PCA, which demonstrated that PCA was able to suppress mesangial cell proliferation in a dose-dependent manner by decreasing the levels of protein expression involved in mesangial expansion including type IV collagen, laminin, and fibronectin [29]. This in vitro evidence reinforces the potential direct action of PRHE at the kidney level in diabetes.

Studies have shown that oxidative stress plays an independent role in the development, progression, and severity of diabetic nephropathy [16,30]. ROS can damage renal cells by oxidizing membrane phospholipids, proteins, carbohydrates, and nucleic acids. ROS are also secondary messengers that activate many signaling cascades, leading to cell damage and deterioration of kidney function in diabetic kidney disease [16,30]. Mitochondria are well recognized as a major source of ROS production. There is evidence linking obesity and diabetes with oxidative stress and mitochondrial dysfunction [30]. The kidneys have high energy demand and are rich in mitochondria, thus mitochondrial dysfunction could contribute to renal oxidative cell injury and consequently renal functional and structural impairments. In line with this view, we detected mitochondrial damage in diabetic rats, as shown by increased ROS production, a dissipation of membrane potential, and a swollen and abnormal mitochondrial ultrastructure. Significant increases in the renal tissue levels of malondialdehyde (a lipid peroxidation product) and decreases in antioxidants (GSH, SOD, GPx) were also observed, indicating renal oxidative injury. These alterations were markedly diminished by PRHE, which is most likely mediated through the mitochondrial protective properties of PCA and C3G. PCA has been demonstrated to protect cardiac and brain mitochondrial dysfunction in streptozotocin-induced diabetic rats [31,32]. The protective role of C3G by modulating intracellular signaling and maintaining mitochondrial function, rather than acting solely as an antioxidant, has also been reported in cardiac ischemia-reperfusion injury [33]. Studies using HK-2 cells further demonstrated that C3G inhibited high glucose-induced ROS production, prevented mitochondrial membrane potential loss, and protected against mitochondrial dysfunction-mediated cell death [34]. Taken together, it is proposed that treatment with PRHE preserves mitochondrial integrity, decreases oxidative stress, and, finally, leads to the protection of diabetic kidney injury. It is interesting to mention here that there was no greater production of mitochondrial ROS (including the kidney tissue levels of MDA) in the metformin-treated diabetic group, though blood glucose level was slightly higher than normal. Beyond its well-known blood glucose regulatory action, metformin has been confirmed to possess antioxidant properties [35]. This action of metformin is mediated through activation of AMPK secondary to its inhibition on the mitochondrial respiratory chain complex 1, the major site of ROS generation in mitochondria [35].

SIRT3, a major mitochondrial deacetylase, was recently highlighted as a novel regulator of mitochondrial function and redox homeostasis [13]. SIRT3 plays a key role in deacetylating and modifying the enzymatic activities of several mitochondrial proteins, including SOD2 [36]. SOD2 is the main antioxidant enzyme in the mitochondria that is responsible for scavenging the superoxide anion, a byproduct of the mitochondrial electron transport chain. This function allows SOD2 to clear mitochondrial ROS, maintain mitochondrial oxidative equilibrium, and confer protection against mitochondrial injury and death [37]. Evidence has suggested that the deacetylation of SOD2 by SIRT3 is necessary for the activation of SOD2, and this deacetylase activity of SIRT3 is further modulated by PGC-1α [13].

Herein, we detected a significant reduction in the expression of PGC-1α and SIRT3 in parallel with the increased Ac-SOD2 and decreased SOD2 expression in diabetic rats. Importantly, PRHE supplementation to the diabetes group was able to normalize these changes. This suggests that the protection against mitochondrial dysfunction and redox imbalance in diabetic nephropathy by PRHE may be a result of the modification of PGC-1α-SIRT3-SOD2 signaling transduction. Consistent with our results, an increased expression ratio of Ac-SOD2/SOD2 followed by increased mitochondrial oxidative stress and mitochondrial dysfunction were observed in Zucker diabetic fatty rats upon reduction of SIRT3 activity [38]. A study of SIRT3 knockout mice demonstrated similar results [36,39]. A recent publication also showed the protection against diabetic kidney injury in BTBR ob/ob mice by honokiol, a polyphenolic compound isolated from magnolia bark, through the maintenance of mitochondrial stability by activating SOD2 and restoring PGC-1α expression [40].

In this study, increased PGC-1α, SIRT3, and SOD2 expressions and decreased Ac-SOD2 expression in diabetic rats treated with a combination of metformin and PRHE were found to be very similar compared to the single therapy with metformin or PRHE. A previous study showed the upregulation of SIRT3 expression followed by a decrease in mitochondrial ROS formation after metformin treatment [41]. There is also a report that activation of PGC-1α is one of the therapeutic mechanisms of metformin in diabetes [42]. As PGC-1α and its subsequent downstream signals appear to be the same target for both metformin and PRHE, this may underlie the lack of additional therapeutic effects in diabetes treated with a combination regimen. However, this issue deserves future exploration.

## 5. Conclusions

This study provides convincing evidence indicating the promising role of PRHE in preventing the development and progression of diabetic nephropathy. The potential of PRHE is apparently associated with its ability to retain mitochondrial integrity and redox equilibrium within the kidney through the activation of the PGC-1α-SIRT3-SOD2 signaling pathway. The outcomes substantiate the worth of this agricultural waste and highlight the opportunity to develop purple rice husk as a dietary supplement or health product, which will be of great value in developing countries with limited resources and a high incidence of diabetes mellitus.

## Figures and Tables

**Table 1:** Effects of purple rice husk extract (PRHE) and metformin on metabolic indexes.

Parameters	NDV	DMV	DMM	DME	DMME
Food intake (g/day)	20.83 ± 0.26 ^a^	17.58 ± 0.53 ^b^	17.57 ± 0.34 ^b^	17.05 ± 0.38 ^b^	17.64 ± 0.49 ^b^
Energy intake (kcal/day)	77.90 ± 0.98 ^a^	82.25 ± 2.46 ^a^	82.22 ± 1.59 ^a^	79.79 ± 1.78 ^a^	82.56 ± 2.30 ^a^
BW gain (%)	39.17 ± 1.81 ^a^	76.22 ± 7.78 ^b^	49.90 ± 3.05 ^a^	46.61 ± 3.36 ^a^	44.48 ± 4.75 ^a^
VF/100 g BW	5.91 ± 0.62 ^a^	13.33 ± 0.69 ^b^	10.60 ± 0.40 ^c^	9.26 ± 0.69 ^c^	10.08 ± 0.56 ^c^
Total cholesterol (mg/mL)	61.19 ± 1.77 ^a^	107.60 ± 3.07 ^b^	63.00 ± 2.17 ^a^	61.92 ± 5.31 ^a^	72.55 ± 8.60 ^a^
Triglycerides (mg/mL)	52.25 ± 4.68 ^a^	103.50 ± 4.47 ^b^	62.51 ± 3.64 ^a^	57.34 ± 2.24 ^a^	65.82 ± 7.64 ^a^
Glucose (mg/dL)	163.77 ± 7.14 ^a^	386.47 ± 43.31 ^b^	233.41 ± 19.18 ^a^	223.82 ± 9.43 ^a^	230.06 ± 29.79 ^a^
Insulin (ng/mL)	1.92 ± 0.15 ^a^	4.14 ± 0.90 ^b^	1.90 ± 0.21 ^a^	1.82 ± 0.30 ^a^	1.94 ± 0.31 ^a^
HOMA-IR	0.78 ± 0.08 a	3.79 ± 0.69 b	1.06 ± 0.09 a	1.01 ± 0.17 a	1.04 ± 0.14 a

Values are the mean ± SD (*n* = 6). NDV: vehicle-treated normal group; DMV: vehicle-treated diabetic group; DMM: metformin-treated diabetic group; DME: PRHE-treated diabetic group; DMME: metformin plus PRHE-treated diabetic group; BW: body weight; VF: visceral fat; HOMA-IR: homeostasis model assessment of insulin resistance. ^a–c^ Mean values with different letters in the same row are significantly different (*p* < 0.05).

**Table 2:** Effects of purple rice husk extract (PRHE) and metformin on renal function.

Parameters	NDV	DMV	DMM	DME	DMME
KW/BW (g/100 g BW)	0.43 ± 0.02 ^a^	0.56 ± 0.01 ^b^	0.44± 0.01 ^a^	0.47 ± 0.03 ^a^	0.48 ± 0.02 ^a^
Serum creatinine (mg/dL)	0.41 ± 0.04 ^a^	0.63 ± 0.03 ^b^	0.45 ± 0.02 ^a^	0.47 ± 0.02 ^a^	0.47 ± 0.02 ^a^
Creatinine clearance (mL/min/g KW)	0.95 ± 0.04 ^a^	0.55 ± 0.03 ^b^	0.97 ± 0.01 ^a^	0.94 ± 0.03 ^a^	0.98 ± 0.03 ^a^
Urine microalbumin (mg/g creatinine)	22.75 ± 4.56 ^a^	83.37 ± 16.62 ^b^	40.40 ± 11.74 ^a^	43.22 ± 16.09 ^a^	41.12 ± 16.11 ^a^

Values are the mean ± SD (*n* = 6). NDV: vehicle-treated normal group; DMV: vehicle-treated diabetic group; DMM: metformin-treated diabetic group; DME: PRHE-treated diabetic group; DMME: metformin plus PRHE-treated diabetic group; KW: kidney weight; BW: body weight. ^a,b^ Mean values with different letters in the same row are significantly different (*p* < 0.05).

## Data Availability

The data presented in this study are available on request from the corresponding author.

## Appendix A

**Table A1:** Nutritional compositions of the experimental diets.

Compositions	Normal Diet		High-Fat Diet	
	g/100 g Diet	% Energy	g/100 g Diet	% Energy
Carbohydrates	56.21	60.11	26.38	22.54
Fat	4.55	10.95	27.89	53.63
Protein	27.06	28.94	28.81	23.93
Vitamin and mineral	6.54	-	9.92	-
Fiber	3.43	-	4.32	-
Total energy (%)		100		100
Caloric value (kcal/g)		3.74		4.68

**Table A2:** Effects of different concentrations of purple rice husk extract (PRHE) on metabolic indexes and renal function (a preliminary study).

Parameters	NDV	DMV	DME 150	DME 300	DME 600
Food intake (g/day)	20.54 ± 0.17	16.87 ± 0.43 *	17.56 ± 0.76 *	17.22 ± 0.56 *	17.00 ± 0.37 *
Energy intake (kcal/day)	76.82 ± 0.62	78.92 ± 1.99 *	82.20 ± 3.55	80.59 ± 2.63	79.57 ± 1.01
BW gain (%)	41.60 ± 1.45	87.49 ± 4.92 *	56.63 ± 3.87 *,^†^	46.14 ± 5.12 ^†^	37.57 ± 4.41 ^†^
Glucose (mg/dL)	161.18 ± 10.98	433.99 ± 48.50 *	283.00 ± 15.51*,^†^	238.24 ± 3.72 *,^†^	260.37 ± 22.51 *,^†^
Insulin (ng/mL)	1.98 ± 0.12	4.57 ± 1.31 *	3.88 ± 0.35 *,^#^	1.92 ± 0.40 ^†^	2.74 ± 0.36 ^†^
HOMA-IR	0.80 ± 0.10	4.53 ± 0.79 *	2.69 ± 0.20 *,^†^,^#^	1.12 ± 0.22 ^†^	1.77 ± 0.35 ^†^
Serum creatinine (mg/dL)	0.45 ± 0.03	0.63 ± 0.02 *	0.55 ± 0.10	0.48 ± 0.03 ^†^	0.52 ± 0.14
Creatinine clearance (ml/min/g KW)	0.97 ± 0.05	0.54 ± 0.03 *	0.63 ± 0.16 *	0.91 ± 0.04 ^†^	0.85 ± 0.15 ^†^

Values are mean ± SD (*n* = 4). NDV: vehicle-treated normal group; DMV: vehicle-treated diabetic group; DME (150, 300, 600): PRHE-treated diabetic group at 150, 300, 600 mg/kg, respectively; BW: body weight; HOMA-IR: homeostasis model assessment of insulin resistance; KW: kidney weight. * *p* < 0.05 vs. NDV, ^†^ *p* < 0.05 vs. DMV, ^#^ *p* < 0.05 vs. DME 300.
